# The Knockout for G Protein-Coupled Receptor-Like PfSR25 Increases the Susceptibility of Malaria Parasites to the Antimalarials Lumefantrine and Piperaquine but Not to Medicine for Malaria Venture Compounds

**DOI:** 10.3389/fmicb.2021.638869

**Published:** 2021-03-15

**Authors:** Benedito M. Santos, Bárbara K. M. Dias, Myna Nakabashi, Celia R. S. Garcia

**Affiliations:** ^1^Department of Clinical and Toxicological Analysis, School of Pharmaceutical Sciences, University of São Paulo, São Paulo, Brazil; ^2^Department of Parasitology, Institute of Biomedical Sciences, University of São Paulo, São Paulo, Brazil

**Keywords:** antimalarials, G protein-coupled receptor-like, medicine for malaria venture compounds, PfSR25, *Plasmodium falciparum*

## Abstract

Previously we have reported that the G protein-coupled receptor (GPCR)-like PfSR25 in *Plasmodium falciparum* is a potassium (K^+^) sensor linked to intracellular calcium signaling and that knockout parasites (PfSR25-) are more susceptible to oxidative stress and antimalarial compounds. Here, we explore the potential role of PfSR25 in susceptibility to the antimalarial compounds atovaquone, chloroquine, dihydroartemisinin, lumefantrine, mefloquine, piperaquine, primaquine, and pyrimethamine and the Medicine for Malaria Venture (MMV) compounds previously described to act on egress/invasion (MMV006429, MMV396715, MMV019127, MMV665874, MMV665878, MMV665785, and MMV66583) through comparative assays with PfSR25- and 3D7 parasite strains, using flow cytometry assays. The IC_50_ and IC_90_ results show that lumefantrine and piperaquine have greater activity on the PfSR25- parasite strain when compared to 3D7. For MMV compounds, we found no differences between the strains except for the compound MMV665831, which we used to investigate the store-operated calcium entry (SOCE) mechanism. The results suggest that PfSR25 may be involved in the mechanism of action of the antimalarials lumefantrine and piperaquine. Our data clearly show that MMV665831 does not affect calcium entry in parasites after we depleted their internal calcium pools with thapsigargin. The results demonstrated here shed light on new possibilities on the antimalarial mechanism, bringing evidence of the involvement of the GPCR-like PfSR25.

## Introduction

Present in more than 90 countries spread across different continents, malaria is still an infectious disease with a high global impact. Epidemiological data released by the WHO estimate there are approximately 228 million cases of the disease, causing more than 400,000 deaths annually, with children under 5 years of age being the group that is most affected by the disease and representing 67% of the total deaths from malaria globally (World malaria report, 2019).

The complex biological cycle of malaria includes different microenvironments in the *Anopheles* mosquito vector, the liver stage and the intraerythrocytic cycle, which helps to hamper the control and eradication of the disease (Koyama et al., 2009; Da Silva et al., 2013; Schuck et al., 2013; Josling and Llinás, 2015). Another important factor that has also contributed to the ineffectiveness of combating malaria is the emergence of strains resistant to the drugs used for the treatment (Khoury et al., 2020; Wicht et al., 2020). The widespread use of antimalarials in recent decades has triggered a selection of parasites, causing *Plasmodium falciparum* to develop drug resistance mechanisms (Le Bras and Durand, 2003; White, 2004).

In an attempt to delay antimalarial resistance, the use of combination therapies, such as the so-called artemisinin-based therapies (ACTs), which include an artemisinin analog in conjunction with another longer-acting compound, has been established (Kremsner and Krishna, 2004). In many cases, resistance is associated with mutations in the genes for carrier proteins such as Plasmodium falciparum chloroquine resistance transporter (PfCRT) and Plasmodium falciparum multidrug resistance 1 (PfMDR1; Reed et al., 2000; Le Bras and Durand, 2003; Lee et al., 2018, Wicht et al., 2020).

However, the mechanisms of action and the factors that lead to drug resistance are not yet clear (Price et al., 2006). The sequencing of the *Plasmodium* genome enabled the acquisition of new information regarding this parasite’s biology, such as the identification of G protein-coupled receptor (GPCR)-like (PfSR1, PfSR10, PfSR12, and PfSR25; Madeira et al., 2008). GPCRs are a large protein family of transmembrane receptors that are involved in the most diverse signal transduction pathways (Alberts et al., 2017; Alhadeff et al., 2018; Pereira et al., 2020). GPCRs represent almost 50% of pharmaceutical company drug targets (Hauser et al., 2017; Sriram and Insel, 2018).

During its complex life cycle, the parasite switches from an environment with a high potassium (K^+^) concentration to a low K^+^ concentration. Previous studies on the characterization of the GPCR-like PfSR25 by Moraes et al. (2017) indicated that the parasite receptor (PfSR25) acts as a K^+^ sensor that is linked to increases in cytosolic calcium (Ca^2+^_cyt_). The Ca^2+^_cyt_ increase is triggered in assays performed on isolated parasites, and the observed Ca^2+^ increase could be blocked by either the phospholipase C (PLC) inhibition or by depleting the internal Ca^2+^ stores. However, when the *pfsr25* gene was deleted, no rise in Ca^2+^_cyt_ was observed in response to the change in K^+^ concentration. The knockout parasite has also been shown to be more susceptible to oxidative stress, amino acid deprivation, and some antimalarial compounds (Moraes et al., 2017). Recently, Santos et al. (2020) reported the susceptibility of the PfSR25 knockout strain to synthetic compounds derived from 1*H*-and 2*H*-1,2,3-triazole ring stereoisomers.

To further characterize the role of PfSR25 in *P. falciparum*, we evaluated the IC_50_ and IC_90_ for the wild-type strain (3D7) and the SR25-knockout strain (PfSR25-), incubating them with the antimalarials atovaquone, chloroquine, dihydroartemisinin, lumefantrine, mefloquine, piperaquine, primaquine, and pyrimethamine. These compounds possess different mechanisms of action to block the *P. falciparum* cell cycle.

We next compared the response of the two *P. falciparum* strains, 3D7 and the GPCR-like PfSR25 knockout, to antimalarial compounds from the Medicine for Malaria Venture (MMV) library (MMV006429, MMV396715, MMV019127, MMV665874, MMV665878, MMV665785, and MMV665831). For these studies, we used flow cytometry and markers for parasite nucleic acid staining, namely, SYBR Green I and MitoTracker Deep Red, to evaluate the mitochondrial membrane potential of the parasites.

In addition, we also investigated the involvement of the promising anti-malarial compound MMV665831, which acts to block the parasite’s egress/invasion (Subramanian et al., 2018), regarding its involvement in store-operated calcium entry (SOCE). For that experiment, wild-type parasites (3D7) were loaded with Fluo-4/AM, and the calcium dynamics were monitored in parasites that had previously been incubated with 1 μM of the MMV665831 compound. Our data show no significant difference in the SOCE assays when the 3D7 parasites were preincubated in the presence of MMV665831 compared with the control, in which using the solvent alone as the compound does not affect calcium entry in parasites. These data reinforce the idea that the secondary messengers, such as cGMP and cAMP, are central to signaling aspects of parasite egress and invasion.

## Materials and Methods

All experiments were carried out following the standard biosafety and institutional safety procedures required by the School of Pharmaceutical Sciences at the University of São Paulo. The handling of *P. falciparum* cultures was carried out in a cell culture facility with biosafety level 2.

### Maintenance of *Plasmodium falciparum* Cultures

Red blood cells infected with *P. falciparum* strain 3D7 and the PfSR25 receptor knockout strain (PfSR25-) were grown according to (Trager and Jensen, 1976). The cultures were kept in 175 cm^2^ culture bottles (Greiner Bio-One) containing RPMI 1640 (Gibco) with 0.5% NaHCO_3_, 0.04% gentamicin sulfate, and 0.05% hypoxanthine and supplemented with 0.5% AlbuMAX I (Gibco). The culture bottles were maintained at 37°C under a mixture of gases consisting of 90% N_2_, 5% O_2_, and 5% CO_2_. The parasitemia was determined from a blood smear stained with Rapid Panotic (Laborclin). PfSR25- parasites were selected with 2.5 μg/ml blasticidin (Sigma-Aldrich).

#### Incubation Tests of Antimalarials in *Plasmodium falciparum* Cultures

To assess the susceptibility of the *P. falciparum* 3D7 and PfSR25- strains to the classic antimalarials, asynchronous cultures of *P. falciparum* (3D7 and PfSR25-) with 0.3% parasitemia and 1% hematocrit were incubated in 96-well plates for 72 h with antimalarial compounds at different concentrations at 37°C under an atmosphere consisting of 90% N_2_, 5% O_2_, and 5% CO_2_. As a control, the parasite culture incubation was performed with Dimethyl sulfoxide (DMSO) solvent at a concentration corresponding to the highest percentage present in the tests.

Incubation was performed with atovaquone (0.019–20 nM), chloroquine (0.24–250 nM), dihydroartemisinin (0.03–40 nM), lumefantrine (0.12–128 nM), mefloquine (0.25–300 nM), piperaquine (0.15–160 nM), primaquine (39.06–40,000 nM), and pyrimethamine (0.24–250 nM).

#### Incubation Assays With MMV Library Compounds

The Malaria Box library received from the MMV (Geneva, Switzerland) contained 390 compounds distributed in five 96-well plates that were stored at −20°C until use. Ten microliters of cell culture-grade DMSO (Sigma-Aldrich) was added to the plates to make 10 mM stocks that were eventually used to prepare the assays. A selection of the compounds presents in the library used in this study was based on the results obtained from the work of Subramanian et al. (2018). The selected compounds (MMV006429, MMV396715, MMV019127, MMV665874, MMV665878, MMV665785, and MMV665831) were incubated with *P. falciparum* cultures as mentioned previously, in section “Incubation Tests of Antimalarials in *Plasmodium falciparum* Cultures.” To determine the IC_50_ values, serial dilutions of the compounds at concentrations ranging from 0.0048 to 5 μM were performed in triplicates over three independent experiments.

#### Parasite Marking and Flow Cytometry Assay

After 72 h of incubation with the compounds, the parasites were dyed with nucleic acid staining SYBR Green I (1X) and MitoTracker Deep Red (50 nM) as a membrane potential marker that tends to deposit on the mitochondrial organelles, and both markers were diluted in PBS (Ekland et al., 2011). After 20 min of incubation at 37°C, the final parasitemia was obtained by reading the plates using a FACSCalibur flow cytometer (Becton Dickinson). The results obtained by cytometer were converted into dot plots and analyzed using FlowJo-V10.7.1 software (FL4-H by FL1-H).

All the tests were performed in triplicates (with an N equal to three independent experiments), and the parasitemia inhibition percentage was calculated in reference to the solvent (DMSO). The dose-response curves and inhibitory concentration values IC_50_ and IC_90_ were calculated using GraphPad Prism-V5.01 software.

### Real-Time Quantitative PCR

*Plasmodium falciparum* 3D7 parasites were synchronized with 5% sorbitol (Lambros and Vanderberg, 1979). Parasites were collected in the trophozoite phase (28–32 h) with approximately 4% parasitemia. Parasites were treated with 100 nM of piperaquine and incubated for 24 h before extraction of the total RNA with Trizol (Invitrogen), 0.01% DMSO was used as control. Purified RNA was quantified in a UV-Vis Nano Drop 2000c (Thermo Scientific). Three independent experiments were carried out in triplicates.

The cDNA synthesis was performed using random primers and reverse transcriptase Superscript II (Invitrogen) according to the manufacturer’s protocol. SYBR Green (Applied Biosystems) was used in quantitative real-time PCR on a 7300 Real-Time PCR System (Applied Biosystems). Amplification was carried out as follows: first step 50°C for 2 min and 95°C for 10 min for enzymatic activation followed by 40 cycles of 55°C for 0.15 min for denaturation and 60°C for 1 min for annealing/extension. The primers used were as follows: PF3D7_0713400 – Fwd GGGGATTCATACCGTTTTCACA and PF3D7_0713400 – Rvs AACAAGGCTAGCAGTTCCCA. Changes in relative expression were determined by 2^(−ΔΔCt)^. We performed statistical analysis with ΔΔCt values with relative expression of the normalized genes for the housekeeping gene seryl-tRNA synthetase.

### Spectrofluorometric Quantifications of Calcium (Ca^2+^)_cyt_ in 3D7 and PfSR25- Strains

The calcium dynamics were evaluated as previously mentioned in (Pecenin et al., 2018). In brief, the parasites were synchronized with 5% sorbitol (Lambros and Vanderberg, 1979) and isolated at the trophozoite stage (28–32 h). They were subsequently loaded with Fluo-4/AM in buffer M (116 mM NaCl, 5.4 mM KCl, 0.8 mM MgSO4, 5.5 mM d-glucose, 50 mM MOPS, and 2 mM CaCl_2_, pH 7.4) containing probenecid 40 μM (Sigma), which was used to prevent the sequestration of Fluo-4/AM in the parasitic digestive vacuole. The parasites were incubated for 1 h at 37°C. The cells were washed three times in buffer M to remove extracellular Fluo4/AM and then incubated with compound MMV665831 (1 μM) for 15 min at 37°C before the agonist was added. Parasites incubated with 0.01% DMSO were used as the control group, in free Ca^2+^ buffer M.

The cytosolic Ca^2+^ dynamics were monitored with excitation at 488 nm and emission at 525 nm using a Shimadzu spectrofluorometer (RF5301PC, Japan), with the parasites (10^7^ cells ml^−1^) being measured in a 1 ml stirred cuvette. All the tests were performed at 37°C, in triplicate, and three independent experiments were performed.

Thapsigargin (5 μM) was added to the buffer (without Ca^2+^), and fluctuations in the [Ca^2+^]_cyt_ were normalized using the baseline fluorescence (F1/F0). SOCE measurements were performed by adding 2 mM Ca^2+^ to the cuvette with free Ca^2+^ buffer and calculated using the F1/F0 equation.

### Statistical Analysis

Three independent experiments were conducted in triplicates for each of the tests mentioned. The GraphPad Prism 5.01 program (GraphPad Software Inc., San Diego, CA, United States) was applied to analyze the data. To evaluate the significant difference between the 3D7 wild-type strain of *P. falciparum* and the knockout strain for the GPCR-like PfSR25 the student’s *t*-test was applied in all the tests performed in this study. The differences were considered statistically different at *p* ≤ 0.05.

## Results

### *Plasmodium falciparum* GPCR-Like PfSR25 Knockout Is More Susceptible to the Antimalarials Lumefantrine and Piperaquine

To understand the role of GPCR-like PfSR25 in parasite susceptibility to antimalarial compounds, we assessed the parasitemia of PfSR25- compared to the wild-type strain 3D7 relative to drugs belonging to the following classes of antimalarials: naphthoquinones (atovaquone), 4-aminoquinolines (chloroquine and piperaquine), endoperoxides (dihydroartemisinin), aryl-amino alcohols (lumefantrine and mefloquine), 8-aminoquinolines (primaquine), and antifolates (pyrimethamine), which are used in first-line malaria treatments.

**Figure 1:**
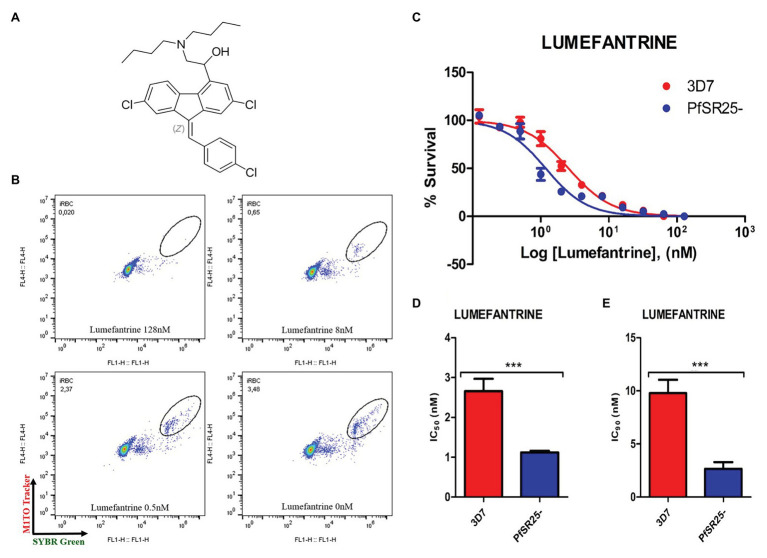
Activity of the antimalarial drug lumefantrine against 3D7 and PfSR25- parasite strains. **(A)** Chemical structure of the antimalarial lumefantrine. **(B)** Dot plots are shown for different concentrations (128 μM, 8 μM, 0.5 μM and control) after 72 h of incubation. **(C)** Survival curves of *Plasmodium falciparum* 3D7 (red) and PfSR25- (blue) in an asynchronous blood stage for lumefantrine with concentrations ranging from 0.12 to 128 nM. The IC_50_ **(D)** and IC_90_ **(E)** values for lumefantrine were evaluated by Student’s *t*-test. The experiments were performed three times independently in triplicate. ^*^Represents the significant difference found using the *t*-test (^***^p ≤ 0.001).

**Figure 2:**
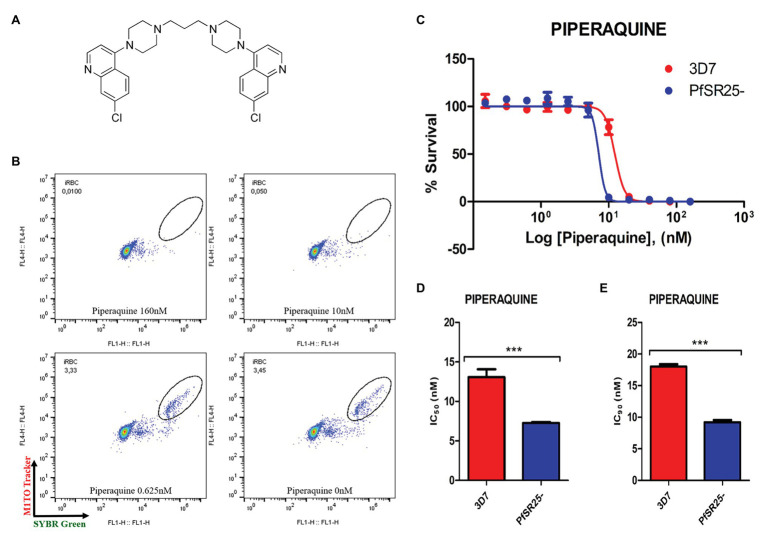
Activity of the antimalarial drug piperaquine in 3D7 and PfSR25- parasite strains **(A)** Chemical structure of the antimalarial piperaquine. **(B)** Dot plots are shown for different concentrations (160, 10, 0.625 μM and control) after 72 h of incubation. **(C)** Survival curves of *P. falciparum* 3D7 (red) and PfSR25- (blue) in an asynchronous blood stage for piperaquine with concentrations ranging from 0.15 to 160 nM. The IC_50_ **(D)** and IC_90_ **(E)** values for piperaquine were evaluated by Student’s *t*-test. The experiments were performed three times independently, in triplicate. ^*^Represents the significant difference found by *t*-test (^***^p ≤ 0.001).

Asynchronous parasites (3D7 and PfSR25-) were incubated with the drugs at different concentrations for 72 h. To assess the final parasitemia, we used flow cytometry and a double-labeled methodology for staining the DNA with SYBR-Green I and labeling the mitochondrial membrane potential using MitoTracker Deep Red to guarantee the analysis of only viable parasites (Ekland et al., 2011). A typical parasitemia analysis is shown in Figures 1, 2. Thus, it was possible to construct survival curves (Supplementary Figure S1) and determine the IC_50_ and IC_90_ values for each compound (Table 1).

**Table 1:** Antimalarial drugs, location of the target(s) and IC_50_ and IC_90_ values for the wild-type strain (3D7) and G protein-coupled receptor (GPCR)-like PfSR25 knockout (PfSR25-).

Antimalarial	Target location (s)	IC_50_ (nM) 3D7	IC_50_ (nM) PfSR25-	IC_90_ (nM) 3D7	IC_90_ (nM) PfSR25-
Atovaquone	Mitochondria (Staines et al., 2018)	0.15 ± 0.03	0.13 ± 0.01	0.70 ± 0.03	0.98 ± 0.26
Chloroquine	Digestive vacuole (Olafson et al., 2015)	11.35 ± 1.98	11.57 ± 0.69	19.10 ± 4.29	25.94 ± 1.14
Dihydroartemisinin	Cytosol and digestive vacuole (Bridgford et al., 2018)	1.77 ± 0.11	1.57 ± 0.54	4.96 ± 1.09	5.17 ± 0.86
Lumefantrine	Cytosol and digestive vacuole (Sullivan, 2017)	2.65 ± 0.30	1.11 ± 0.04	9.77 ± 1.27	2.66 ± 0.62
Mefloquine		9.55 ± 0.44	10.90 ± 2.43	17.43 ± 1.01	22.25 ± 1.58
Piperaquine	Digestive vacuole (Dhingra et al., 2017)	13.07 ± 1.01	7.27 ± 0.13	18.02 ± 0.37	9.20 ± 0.33
Primaquine	Unknown	2,310 ± 180	1,950 ± 330	13,730 ± 1,140	12,050 ± 1,370
Pyrimethamine	Cytosol (Heinberg and Kirkman, 2015)	12.37 ± 1.11	12.06 ± 0.48	28.22 ± 1.48	25.47 ± 0.89

Statistical analysis using Student’s *t*-test was performed to compare the IC_50_ and IC_90_ (±SE) values obtained for the knockout strain (PfSR25-) to those of the wild-type strain (3D7). We observed a significant difference for the antimalarials lumefantrine (Figure 1) and piperaquine (Figure 2), for both the IC_50_ and the IC_90_ values in the assays comparing the knockout strain (PfSR25-) parasites with the wild-type strain (3D7).

For the antimalarial lumefantrine (Figure 1), the IC_50_ and IC_90_ values for the 3D7 strain were 2.65 ± 0.30 nM and 9.77 ± 1.27 nM, respectively, while the knockout strain for PfSR25 showed an IC_50_ of 1.11 ± 0.04 nM and an IC_90_ of 2.66 ± 0.62 nM. Thus, the antimalarial drug displayed reductions of 58.12 and 72.78% for the IC_50_ and IC_90_ values, respectively, in the knockout strain (PfSR25) compared to the values of the wild-type strain (3D7), suggesting greater knockout strain susceptibility to lumefantrine.

For the antimalarial piperaquine (Figure 2), we obtained an IC_50_ value of 13.07 ± 1.01 nM and an IC_90_ of 18.02 ± 0.37 for the wild-type strain 3D7 parasites. When similar experiments were performed with the PfSR25- knockout strain, we found an IC_50_ of 7.27 ± 0.13 nM and an IC_90_ of 9.20 ± 0.33 nM. Thus, we observed reductions of 44.38 and 48.95% for the IC_50_ and IC_90_ values, respectively, in the comparative assays on the SR25 knockout and 3D7 strains.

### *pfsr25* Expression Is Reduced in the Presence of Lumefantrine and Piperaquine

Mwai et al. (2012) reported that the lumefantrine resistant strain generated in the laboratory under the drug pressure showed a downregulation of the *pfsr25* gene in *P. falciparum* the blood stage. Hence, we investigated whether piperaquine would be able to modulate the gene expression of the serpentine receptor GPCR-like PfSR25 from *P. falciparum*.

**Figure 3:**
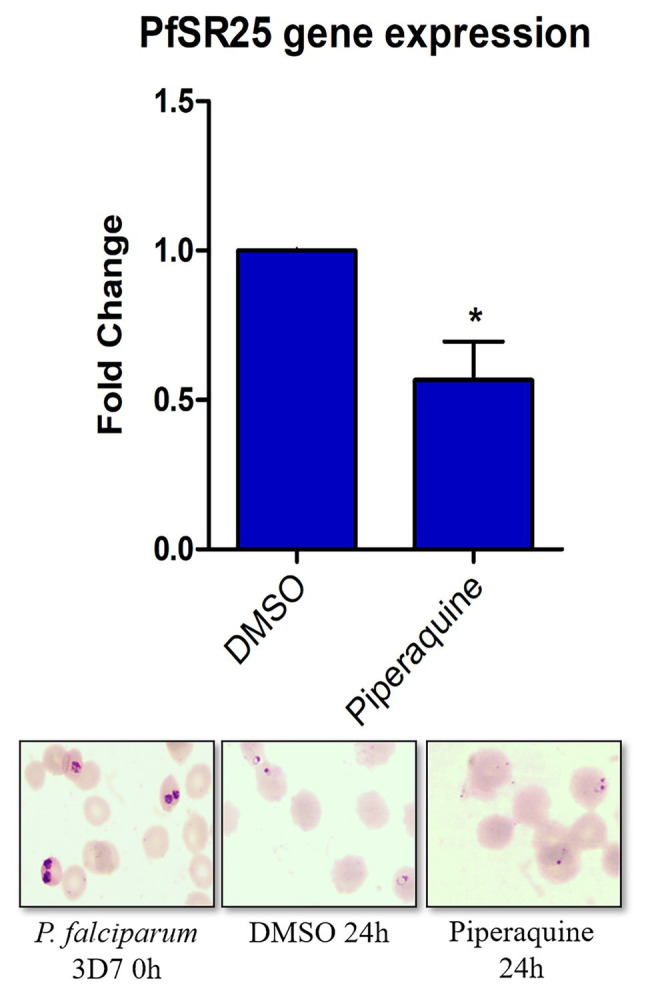
Effect of the antimalarial piperaquine on the expression of the *pfsr25* gene in *P. falciparum* 3D7. Erythrocytes infected with *P. falciparum* parasites in the trophozoite stage were treated with 100 nM piperaquine for 24 h. The expression of *pfsr25* was analyzed and compared with the parasites that received only the solvent. The gene expression was normalized for the housekeeping gene Seryl-tRNA synthase. Three independent experiments were performed in triplicates. ^*^Indicates a significant difference in gene expression by the Student’s *t*-test (^*^p ≤ 0.05).

*Plasmodium falciparum* 3D7 parasites synchronized in the trophozoite stage (28–32 h) were treated with 100 nM piperaquine for 24 h. Figure 3 shows that the parasites submitted to antimalarial high-pressure medium show a decrease in the expression of *pfsr25*. In our results, we observed that the antimalarial piperaquine (100 nM) presence leads to a 43.3% decrease in the expression of *pfsr25* compared to the control. Thus, our data corroborate with Mwai et al. (2012) regarding the role of lumefantrine and piperaquine to interfere in the gene expression of PfSR25.

### PfSR25 Knockout Strain Is Not Susceptible to MMV Compounds That Act on Parasite Egress/Invasion to Red Blood Cells

Following the investigation on the susceptibility of 3D7 and PfSR25- parasites to the classical antimalarials, we evaluated the IC_50_ and IC_90_ values for seven compounds from the MMV drug library, as shown in Table 2. The compound selection was based on the results by Subramanian et al. (2018), in which the authors identified compounds that target the egress/invasion of the intraerythrocytic cycle in the malaria parasite *P. falciparum*.

**Table 2:** Compounds from the Medicine for Malaria Venture (MMV), with chemical structures and IC_50_ and IC_90_ values for the wild-type strain (3D7) and PfSR25- parasitess.

Compound	Chemical structure	IC_50_ (μM) 3D7	IC_50_ (μM) PfSR25-	IC_90_ (μM) 3D7	IC_90_ (μM) PfSR25-
MMV006429	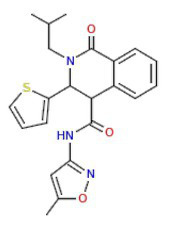	2.34 ± 0.26	2.13 ± 0.10	3.30 ± 0.27	3.14 ± 0.36
MMV396715	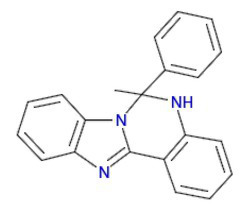	1.48 ± 0.06	1.30 ± 0.08	2.25 ± 0.07	2.23 ± 0.14
MMV019127	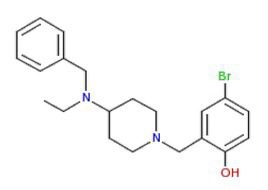	0.86 ± 0.08	0.77 ± 0.08	1,73 ± 0.16	1.78 ± 0.10
MMV665874	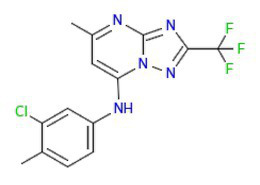	0.33 ± 0.04	0.33 ± 0.01	0.70 ± 0.08	0.71 ± 0.09
MMV665878	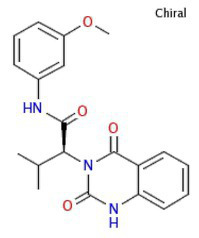	0.17 ± 0.20	0.19 ± 0.21	0.26 ± 0.003	0.29 ± 0.011
MMV665785	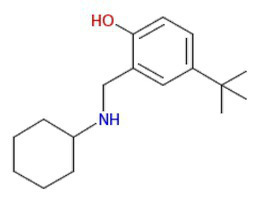	0.082 ± 0.04	0.094 ± 0.06	0.20 ± 0.0015	0.19 ± 0.0047
MMV665831	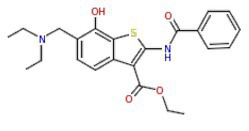	0.087 ± 0.001	0.065 ± 0.007	0.14 ± 0.003	0.13 ± 0.006

The images of the chemical structures for each compound were taken from the MMV database (About the Malaria Box | Medicines for Malaria Venture, n.d.).

**Figure 4:**
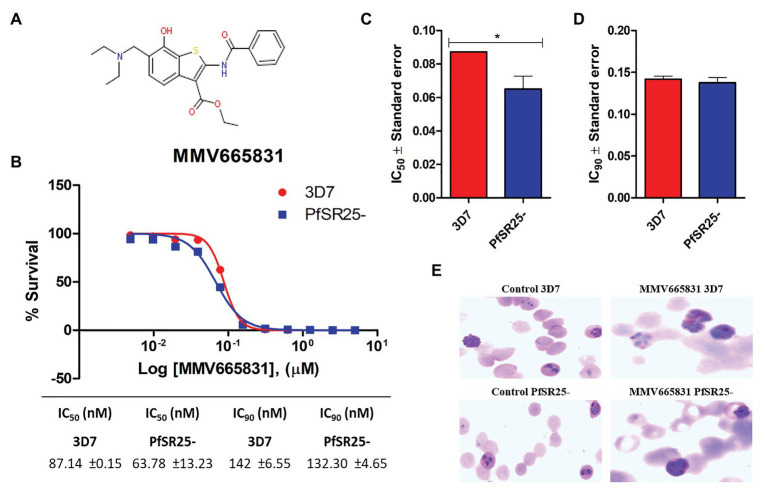
Determination of MMV665831 antiplasmodial activity in strains 3D7 (red) and PfSR25- (blue). **(A)** Chemical structure of the MMV665831 compound. **(B)** Dose-response curve, with concentrations varying from 0.0048 to 5 μM. The IC_50_ **(C)** and IC_90_ **(D)** values were calculated using GraphPad Prism software and the values were compared between the 3D7 and PfSR25- parasite strains by Student’s *t*-test. **(E)** Microscopic image of Giemsa-stained blood smears, parasites (3D7 and PfSR25-) were incubated with compound MMV665831 (0.3125 μM) for 72 h, parasites in the control group were incubated with 0.01% Dimethyl sulfoxide (DMSO). The experiments were performed three independent times in triplicate. ^*^Represents the significant difference found by *t*-test (^*^p ≤ 0.05).

The results obtained here demonstrated that the compounds MMV665785 and MMV665831 have IC_50_ values below 100 nM for both the 3D7 strain and the knockout strain. The data do not point to a greater susceptibility to the compounds in the knockout when compared to the wild-type parasites. However, parasites treated with MMV665831 showed a slightly lower IC_50_ when comparing the IC_50_ value of the wild-type strain-with the IC_50_ value of the PfSR25-. Morphological analysis of the parasites, with Giemsa-stained blood smears, showed no difference in the action of this compound in both strains (Figure 4).

### Compound MMV665831 Does Not Interfere in the SOCE Mechanism in *Plasmodium falciparum*

Calcium signaling plays an essential role during the parasite cycle, primarily in the differentiation, motility, egress, and invasion of *Plasmodium* in erythrocytes (Alves et al., 2011; Lourido and Moreno, 2015). SOCE is activated by the depletion of the intracellular calcium stores and is mediated by the grouping of STIM proteins in the endoplasmic reticulum (ER) adjacent to the Orai channels of the plasma membrane that allow the entry of Ca^2+^ into the cells (Broad et al., 2001; Putney, 2003; Hewavitharana et al., 2007; Pecenin et al., 2018).

**Figure 5:**
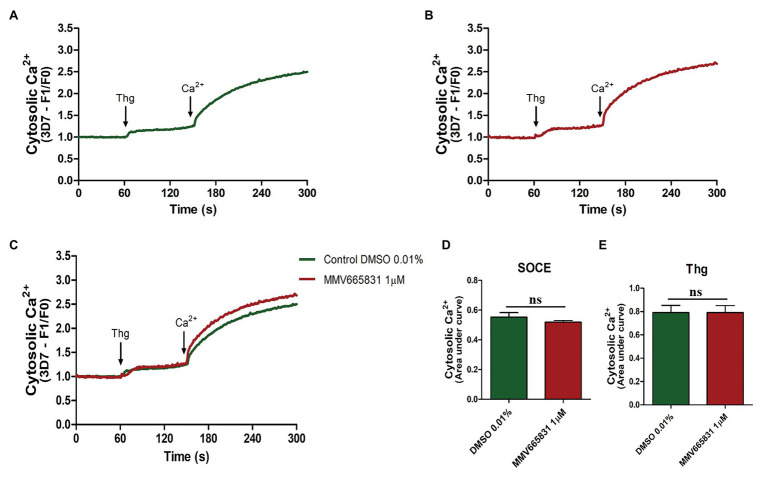
Spectrofluorometric evaluation of the store-operated calcium entry (SOCE) mechanism. Synchronized *P. falciparum* 3D7 in the trophozoite stage (28–32 h) were labeled with Ca^2+^ Fluo-4/AM probe. **(A)** The parasites were previously incubated with DMSO solvent (0.01%). **(B)** The parasites were previously treated with MMV665831 compound (1 μM). After 15 min of treatment, parasites at 10^7^ cells/ml were placed in free Ca^2+^ buffer. Thapsigargin (Thg; 5 μM) was used to deplete the Ca^2+^ from the endoplasmic reticulum (ER). CaCl_2_ (2 mM) was added and the increase in the [Ca^2+^]cyt was monitored. **(C)** Representative curve for SOCE measurement at the control and MMV665831 treated parasites. **(D)** F0 corresponds to the mean of the fluorescence obtained between 140 and 150 s, and F1 corresponds to the mean of the fluorescence obtained between 290 and 300 s. **(E)** F0 corresponds to the mean of the fluorescence obtained between 50 and 60 s, and F1 corresponds to the mean of the fluorescence obtained between 140 and 150 s. Three independent experiments were performed in triplicates. ^*^Represents significant difference found by Student’s *t*-test (^*^p ≤ 0.05).

**Figure 6:**
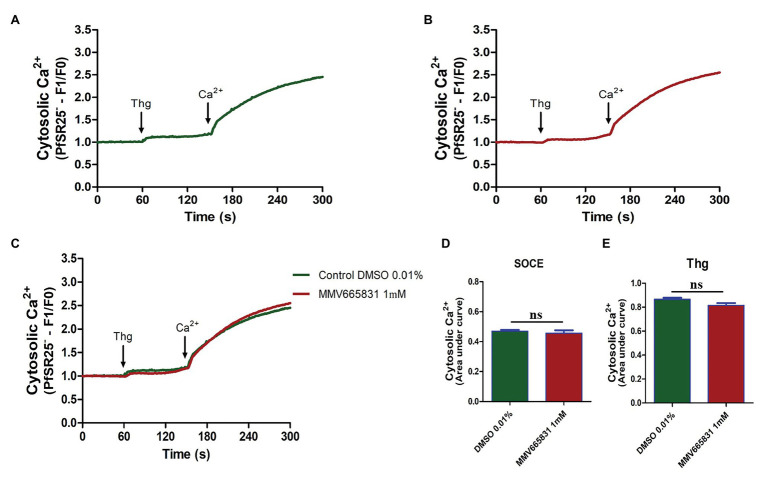
Spectrofluorometric evaluation of the SOCE mechanism. Synchronized *P. falciparum* PfSR25- in the trophozoite stage (28–32 h) were labeled with Ca^2+^ Fluo-4/AM probe. **(A)** The parasites were previously incubated with DMSO solvent (0.01%). **(B)** The parasites were previously treated with MMV665831 compound (1 μM). After 15 min of treatment, parasites at 10^7^ cells/ml were placed in free Ca^2+^ buffer. Thg (5 μM) was used to deplete the Ca^2+^ from the ER. CaCl_2_ (2 mM) was added and the increase in the [Ca^2+^]cyt was monitored. **(C)** Representative curve for SOCE measurement at the control and MMV665831 treated parasites. **(D)** F0 corresponds to the mean of the fluorescence obtained between 140 and 150 s, and F1 corresponds to the mean of the fluorescence obtained between 290 and 300 s. **(E)** F0 corresponds to the mean of the fluorescence obtained between 50 and 60 s, and F1 corresponds to the mean of the fluorescence obtained between 140 and 150 s. Two independent experiments were performed in triplicates. ^*^Represents the significant difference found by *t*-test (^*^p ≤ 0.05).

Thus, we investigated whether the promising antimalarial compound MMV665831 is interfering with the *P. falciparum* SOCE mechanism. For these experiments, parasites at the trophozoite stage were labeled with Fluo-4/AM, and spectrofluorometric analyses were performed to evaluate the SOCE. Figure 5 (3D7 strain) and Figure 6 (PfSR25- strain) show the fluorescence of the isolated parasites in solution within a cuvette, in the absence of external calcium. Adding thapsigargin (Thg; 5 μM) led to ER-calcium depletion and raised the parasite cytosolic calcium in both parasite controls, and the cultures were treated with 1 μM MMV665831 for 15 min. The data show that Thg addition led to the same amount of calcium release under both conditions following Thg addition, and CaCl_2_ was added to reach a (2 mM) final concentration. This procedure is thought to induce SOCE in *P. falciparum* as described by Pecenin et al. (2018).

We then tracked the calcium measurements with Fluo-4/AM-loaded parasites that were previously treated with MMV665831, which affects parasite egress/invasion to red blood cells (Subramanian et al., 2018). For that purpose, we performed experiments similar to those described in Figures 5A, 6A. Therefore, the presence of SOCE were accessed after incubating the parasites at the trophozoite stage (3D7 and PfSR25-), with 1 μM of MMV665831 compound for 15 min, after they had previously been labeled with Fluo-4/AM. The spectrofluorometric data are shown in Figures 5B, 6B, the analysis used to evaluate the presence of SOCE are shown in Figures 5D, 6D, and the analysis on the calcium content depleted by Thg is reflected by the area of the peaks (Figures 5E, 6E).

The data presented here showed that parasites at the trophozoite stage (3D7 and PfSR25-) and pretreated with compound MMV665831 (1 μM) did not display any significant differences when compared to the control group, indicating that this compound does not interfere with the SOCE mechanism of the malaria parasite *P. falciparum*.

## Discussion

The emergence of parasites resistant to classical antimalarial drugs from different compound classes (Ashley et al., 2014; Hanboonkunupakarn and White, 2016; Blasco et al., 2017) demonstrates the urgent need to search for new targets in developing drugs with a different mechanism of action.

G protein-coupled receptors represent a class of receptors with seven transmembrane domains and are the targets for most drugs (Hauser et al., 2017). Four candidates presenting seven transmembrane domains were identified in the malaria parasite *P. falciparum* genome as serpentine-like receptors (Madeira et al., 2008). Among the four candidates (PfSR1, PfSR10, PfSR12, and PfSR25), PfSR25 was identified as a potassium sensor, and parasites knockout were more susceptible to toxic agents such as oxidative stress induced by Nitric Oxide (NO) production and also to stress induced by the removal of albumax from the medium (Moraes et al., 2017).

Our results point that piperaquine and lumefantrine were more active against parasites lacking PfSR25, with significant reductions of 58.12 and 44.38% in the IC_50_ values in PfSR25-, respectively. Piperaquine is concentrated in the digestive vacuole where it is protonated and cannot diffuse out of this compartment through the membrane. These protonated forms bind to the growing hemozoin crystals (Wicht et al., 2020).

The lumefantrine mechanism of action is still yet to be completely elucidated; although it has been implicated in the detoxification of products of heme degradation (Cowell and Winzeler, 2019). Mwai et al. (2012) reported that the lumefantrine-resistant strain has a negative regulation of the *pfsr25* gene in the ring stage. Our results demonstrated that the antimalarial piperaquine is also able to promote a downregulation in the ring stage *pfsr25* gene. Both works imply the involvement of PfSR25 in the mechanism of action of these antimalarials.

Previous studies have shown the importance of the K^+^ shift at the moment of parasite egress/invasion. During hepatic cell migration, sporozoites are exposed to major changes in the K^+^ concentration (Kumar et al., 2007). Kumar et al. (2007) reported that the incubation of sporozoites with K^+^ generated an 8–10-fold increase in the infectivity of *Plasmodium berghei* and a 4–5-fold increase in the infectivity of *Plasmodium yoelii* (Kumar et al., 2007). Singh et al. (2010) reported that exposing merozoites to low K^+^ concentrations provide an external signal that leads to an increase in the Ca^2+^_cyt_, which triggers the translocation of microneme proteins, such as the erythrocyte-binding antigen (EBA175 antigen) and apical membrane antigen-1 (AMA1), which are expressed on the surface of the merozoite.

Kumar et al. (2007) and Singh et al. (2010) reported the importance of K^+^ for the parasite mainly in its hepatic phase. Although the knockout parasite for PfSR25 did not show changes in its intra-erythrocyte cycle *in vitro*, the knockout strain for SR25 in *P. berghei* (PbSR25) was unable to develop a successful infection *in vivo* (Moraes et al., 2017).

To better understand the relationship between PfSR25 and the events that govern egress/invasion in the intra-erythrocytic cycle, we used compounds from MMV (Malaria box) previously described to impair the egress/invasion of the intra-erythrocytic cycle of *P. falciparum* (Subramanian et al., 2018). The results obtained here show that MMV665785 and MMV665831, which were previously described as inhibitors of egress/invasion, presented IC_50_ values below 100 nM.

Previous studies showed that MMV665831 blocked the rupture of infected erythrocytes at nanomolar concentrations and promoted almost a 90% decrease in ring formation at different concentrations (0.3, 1, 3, and 10 μM). The MMV665785 compound was recently reported to impair the rupture of infected erythrocytes and prevent the infection of new ones. Nonetheless, compound withdrawal after 2 h of incubation resulted in normal egress, but compound MMV665831 showed irreversible action, even after withdrawal (Subramanian et al., 2018).

Our results showed that among the tested compounds, MMV665785 and MMV665831 presented higher antimalarial activity *in vitro* with IC_50_ values of 0.082 ± 0.04 μM (82 nM) and 0.087 ± 0.001 μM (87 nM), respectively. Compounds that were previously described to impair egress/invasion did not present higher activity against parasites lacking PfSR25. Only the compound MMV665831 resulted in a lower IC_50_ value in the knockout strain. However, we observed no difference regarding the concentration needed to eliminate 90% (IC90) of the parasite population between the two strains (3D7 and PfSR25).

Calcium is an intracellular messenger that is present in malaria parasites, and it plays key roles in the parasite during the asexual stages (Brochet and Billker, 2016; Singh et al., 2020). The second messenger Ca^2+^ was found to participate in erythrocyte invasion (Cowman and Crabb, 2006). Weiss et al. (2015) observed that in Ca^2+^-free media, the invasion rate decreases significantly, although the merozoites could attach to new erythrocytes.

Moreover, calcium also participates directly in the activation of proteins that participate in key signaling pathways, such as protein kinases. Receptors for activated C kinases are scaffold proteins that anchor several signaling proteins and are involved in modulating the cell cycle. Despite the absence of classical PKC from the *Plasmodium* database, PfRACK has been identified (Madeira et al., 2003). Moreover, Ca^2+^ mobilization regulates important processes in protist biology (Cruz et al., 2012; Borges-Pereira et al., 2015).

The calcium-dependent protein kinase signaling pathway has been identified in the *Plasmodium* database (Ward et al., 2004; Doerig et al., 2008) and controls few processes in the malaria parasite. One of these processes is male gamete formation and zygote development (Reininger et al., 2005), but the molecular mechanisms of calcium release remain poorly understood.

In addition, Ca^2+^ participates in other important pathways such as the pathway triggered by tryptophan derivatives. Melatonin activates phospholipase C (PLC), resulting in IP3 production and the release of Ca^2+^ from the ER (Passos and Garcia, 1997; Alves et al., 2011; Garcia et al., 2017). Furthermore, *P. falciparum* treated with melatonin presented an increase in cAMP levels that was blocked by the PLC inhibitor U73122. Moreover, treating with cAMP analog 6-Bz-cAMP causes a Ca^2+^_cyt_ increase that is dependent on the PKA, suggesting crosstalk between Ca^2+^ and cAMP (Beraldo et al., 2005; Gazarini et al., 2011). The PLC-IP3 mechanism is thought to be fundamental to the SOCE mechanism, the stored-operated Ca^2+^ entry mechanism activated by the depletion of the intracellular calcium stores (Putney, 2003) that is present in *P. falciparum* (Pecenin et al., 2018).

In this study, we have shown that compound MMV665831, an inhibitor of egress/invasion, does not significantly interfere in the store-operated Ca^2+^ entry in *P. falciparum* 3D7 and PfSR25- at the trophozoite stage. The same result was observed when the experiment was performed on the knockout strain for the GPCR-like PfSR25. Our results suggest that this compound action in the invasion process does not occur through the store operated Ca^2+^ entry and that PfSR25 is not necessary for the SOCE mechanism to occur.

During egress/invasion, the parasite experiences a K^+^ shift (Kumar et al., 2007), and the PfSR25 is involved in sensing the K^+^ alterations in the environment. The results here show that *P. falciparum* receptor PfSR25 is not involved in the mechanism of action of MMV drugs known to block the egress/invasion of the human’s intraerythrocytic cycle malaria parasite *P. falciparum*.

## Conclusion

The results show that parasites lacking the PfSR25 receptor are more susceptible to lumefantrine and piperaquine, classical antimalarials that act on the digestive vacuole with heme metabolism. However, we found no difference between the 3D7 and PfSR25- strain after incubating parasites for 72-h with atovaquone, dihydroartemisinin, mefloquine, primaquine, pyrimethamine, and chloroquine at the drug range concentration 0.24–250 nM.

The studies on MMV compounds lead us to conclude that PfSR25 is not involved in the action of compounds that inhibit the egress/invasion process, although further efforts are necessary to elucidate the participation of the receptor in these processes. Our data indicate the potential involvement of GPCR-like PfSR25 in the mechanism of action of lumefantrine and piperaquine in the malaria parasites *P. falciparum* and points the GPCR-like as a novel tool to study antimalarial drugs.

## Data Availability Statement

The raw data supporting the conclusions of this article will be made available by the authors, without undue reservation.

## Author Contributions

BS, MN, and BD conducted the experiments. BS and CG designed the experiments. CG supervised all the work. BS, BD, and CG wrote the manuscript. All authors contributed to the article and approved the submitted version.

### Conflict of Interest

The authors declare that the research was conducted in the absence of any commercial or financial relationships that could be construed as a potential conflict of interest.
