# Supplementary material for: The Knockout for G Protein-Coupled Receptor-Like PfSR25 Increases the Susceptibility of Malaria Parasites to the Antimalarials Lumefantrine and Piperaquine but Not to Medicine for Malaria Venture Compounds
Source: Front Microbiol. 2021 Mar 15;12:638869. doi: 10.3389/fmicb.2021.638869 (PMC8006397; doi:10.3389/fmicb.2021.638869)
Supplement: Supplementary file 1 [file Data_Sheet_1.pdf]

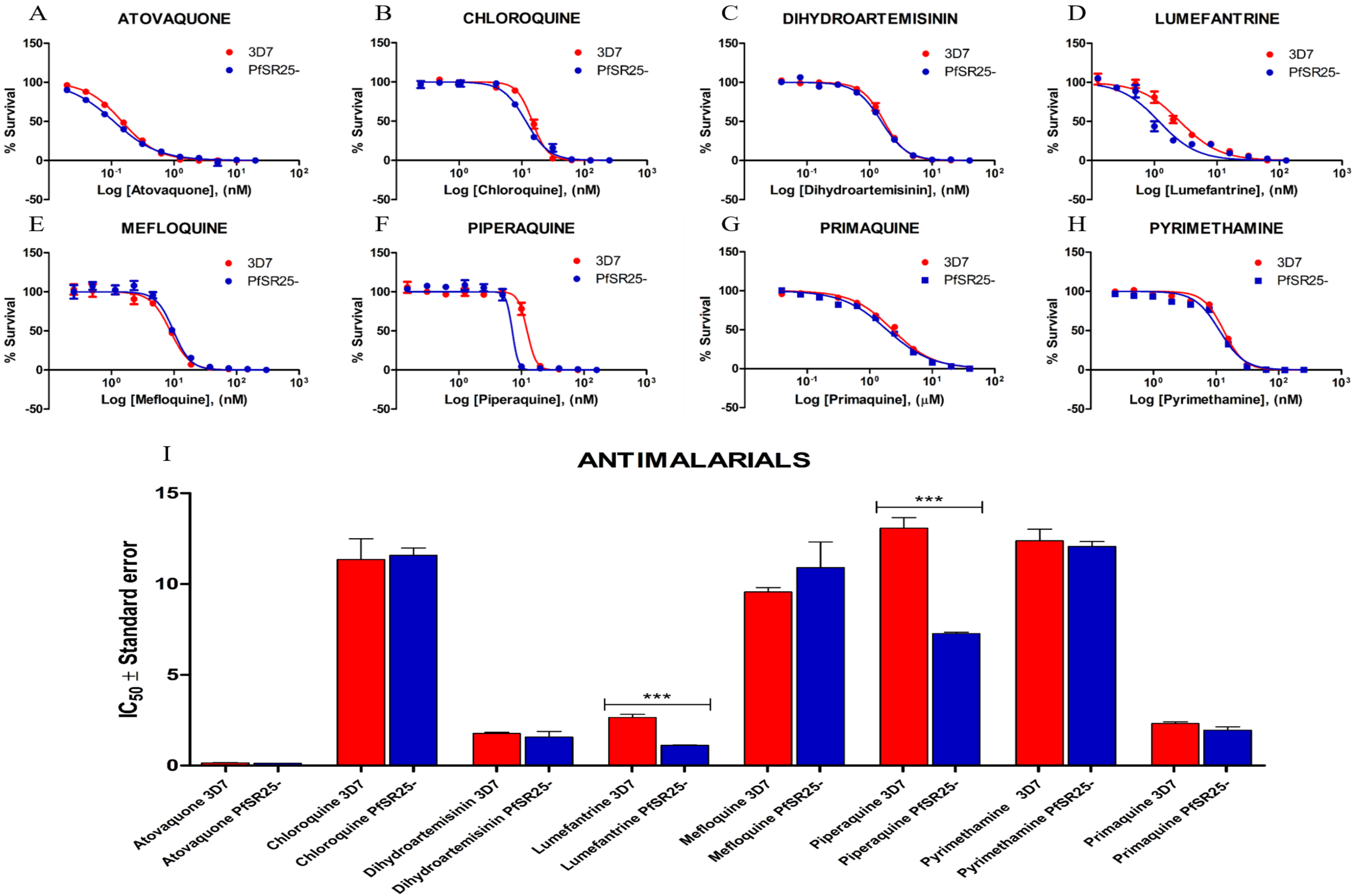

Supplementary Figure 1: Dose-response curves, Erythrocytes infected with *P. falciparum* 3D7 (Red) or PfSR25- (Blue) were incubated for 72 h with antimalarial compounds (**A**) Atovaquone, (**B**) Chloroquine, (**C**) Dihidroartemisinin, (**D**) Lumefantrine, (**E**) Mefloquine, (**F**) Piperaquine, (**G**) Primaquine and (H) Pyrimethamine. (**I**) Statistical calculation of the difference between 3D7 and PfSR25- strains on the IC_{50} values taking into account the mean error (±) calculated by Student's t-test.
