# Supplementary material for: The Knockout for G Protein-Coupled Receptor-Like PfSR25 Increases the Susceptibility of Malaria Parasites to the Antimalarials Lumefantrine and Piperaquine but Not to Medicine for Malaria Venture Compounds
Source: Front Microbiol. 2021 Mar 15;12:638869. doi: 10.3389/fmicb.2021.638869 (PMC8006397; doi:10.3389/fmicb.2021.638869)
Supplement: Supplementary file 2 [file Data_Sheet_2.pdf]

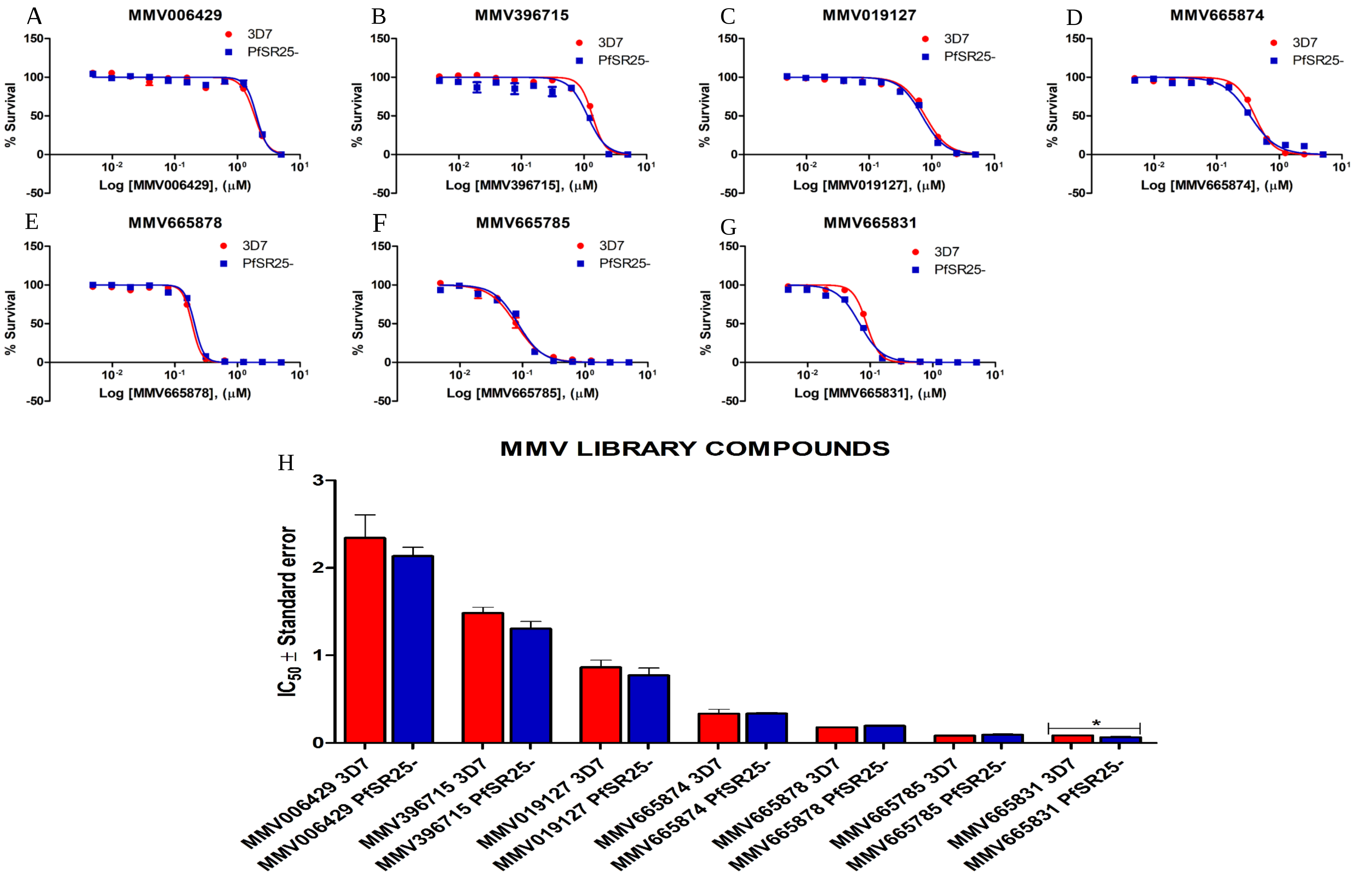

Supplementary Figure 2: Dose-response curves, Erythrocytes infected with *P. falciparum* 3D7 (Red) or PfSR25- (Blue) were incubated for 72 h with MMV compounds (**A**) MMV006429, (**B**) MMV396715, (**C**) MMV019127, (**D**) MMV665874, (**E**) MMV665878, (**F**) MMV665785 and (**G**) MMV665831. (**H**) Statistical calculation of the difference between 3D7 and PfSR25- strains on the IC_{50} values taking into account the mean error (±) calculated by Student's t-test.
